# Bioavailable fluoride in calcium-containing dentifrices

**DOI:** 10.1038/s41598-020-80503-x

**Published:** 2021-01-08

**Authors:** Peiyan Shen, James R. Fernando, Yi Yuan, Glenn D. Walker, Coralie Reynolds, Eric C. Reynolds

**Affiliations:** Centre for Oral Health Research, Melbourne Dental School, Bio21 Institute, The University of Melbourne, 720 Swanston Street, Carlton, Melbourne, VIC 3010 Australia

**Keywords:** Oral diseases, Biochemical assays

## Abstract

Calcium added to dentifrices can complex with fluoride ions to reduce intra-oral bioavailability and therefore efficacy in preventing dental caries. Six commercially available dentifrices containing different types of calcium and fluoride were analyzed for total and bioavailable fluoride levels by adding 10 g of dentifrice to 30 mL of distilled deionized water and mixing vigorously for 1 min to simulate toothbrushing. One milliliter of the dentifrice/water slurry was immediately centrifuged and the supernatant removed for bioavailable fluoride analysis and the mixed slurry prior to centrifugation used for total fluoride analysis using a modified microdiffusion method. The concentration of fluoride was determined using a fluoride ion-selective electrode calibrated with internal fluoride standards. All the dentifrices had similar total fluoride concentrations to those indicated on their labels (94% to 105%). However, only one dentifrice that contained calcium in the form of casein phosphopeptide amorphous calcium phosphate (CPP-ACP) had almost 100% (97%) of fluoride in bioavailable form. The other dentifrices contained calcium carbonate and they exhibited significantly (p < 0.001) lower bioavailable fluoride levels (27% to 61%), through the generation of poorly soluble fluoride phases. The saliva biomimetic CPP, as CPP-ACP, in a dentifrice stabilised calcium and fluoride ions to maintain fluoride’s bioavailability.

## Introduction

Dental caries (tooth decay) is a widespread disease affecting billions of people worldwide^1^. It is characterized by demineralization of tooth mineral (hydroxyapatite) by organic acids produced during dental plaque bacterial fermentation of dietary carbohydrates^2^. The relationship between fluoride (F) and the prevention of dental caries has been studied extensively^3,4^. Frequent exposure to F is considered to be the most effective intervention for the prevention of dental caries^5^. F inhibits demineralization through the process of remineralization of tooth structure with fluorohydroxyapatite^4^. F is added to a variety of oral care products and F-containing dentifrices (toothpastes) are an effective form of F delivery for caries prevention^5–7^.

The regular use of F dentifrices has been associated with a decline in dental caries prevalence in both developed and developing countries, and the anticariogenic efficacy of F within dentifrices has been shown to be dose-dependent^6,8–10^. However, the labelled F content of a dentifrice is not an accurate indicator of its anticariogenic potential^7^. There have been reports of commercially available dentifrices containing less total F than declared and/or releasing relatively low levels of water soluble (bioavailable) F^11–15^. Dentifrices of equal total F concentration can significantly differ in their capacity to release bioavailable F due to differences in their formulation composition^16,17^. Hence, instead of total F content, the amount of bioavailable F released from a dentifrice under conditions of simulated toothbrushing is regarded as a more appropriate predictor of anticariogenic potential^7^.

To maximise bioavailability of F, the dentifrice formulation should not impede F release into saliva during the time of tooth brushing or post-brushing^10,15,18–21^. Furthermore, the intraoral F release from any dentifrice formulation should be accurately reflected through laboratory assessment under simulated conditions to enable quality control^7,22^. Accurate determination of bioavailable F in dentifrices is vital to ensure that the public is protected from formulations with compromised efficacy or no efficacy. Differences in dentifrice matrix components and F compound affect not only soluble F release in vivo, but also the accuracy of various methods for laboratory determination of total and bioavailable F^12,23^. Calcium compounds (e.g. calcium carbonate and calcium phosphates) are now routinely added to dentifrice formulations to enhance abrasive cleaning and release calcium ions to help promote remineralization^24–27^. Remineralization of early caries lesions by F to form fluorohydroxyapatite is calcium-dependent and can become calcium limited such that the addition of stabilised and bioavailable calcium ions to dentifrice formulations has been shown to enhance the ability of F to remineralize tooth enamel^25^. Some of the calcium compounds added to dentifrices are poorly soluble, e.g. calcium carbonate (CaCO_3_), dicalcium phosphate dihydrate (CaHPO_4_·2H_2_O), and tricalcium phosphate (Ca_3_(PO_4_)_2_), and are included in part to enhance the physical removal of plaque during brushing^19^. However, some of these poorly soluble calcium-based abrasives have the capacity to bind (adsorb) F and subsequently inhibit in vivo and/or ex vivo F release and/or measurement^19^. Common F compounds added to dentifrices such as sodium fluoride (NaF), sodium monofluorophosphate (MFP), or stannous fluoride (SnF_2_) have heterogeneous sensitivity to laboratory methods used for measurement of potentially bioavailable F^7,28^. The commonly used method, the fluoride-ion selective electrode (F-ISE), can be adversely affected by certain dentifrice matrix components, and is insensitive to non-ionic, pro-fluoride compounds (e.g. MFP)^7,22^. To overcome these challenges, F detection using F-ISE following an acid hydrolysis and HF microdiffusion method has been used effectively for a wide range of dentifrice formulations^7,29,30^. This method utilizes hexamethyldisiloxane (HMDS) to promote rapid diffusion of F as HF, separating it from dentifrice matrix components to capture the ion in a KOH solution to allow accurate detection with a F-ISE^7^.

As the use of calcium compounds, in particular calcium carbonate, is widespread in commercial dentifrices and these compounds may reduce the level of bioavailable F and thus the dentifrice’s anticariogenic efficacy^22^, the aim of this study was to determine the bioavailable F concentration of different calcium-containing dentifrices using a laboratory procedure simulating toothbrushing^7^ with a modification of the Taves microdiffusion method for F measurement^29^. The measured concentrations of total F and bioavailable F were compared with the manufacturer’s stated F concentrations. The null hypothesis for the study was that all the dentifrices had no difference between bioavailable F and total F.

## Materials and methods

### Dentifrices

Six commercially available dentifrices containing various types of F and calcium were tested (Table 1). The type of F and calcium compound contained in the six dentifrices were: (1) MI PASTE ONE (MPO) 1100 ppm F as NaF and casein phosphopeptide-stabilised amorphous calcium phosphate (CPP-ACP); (2) COLGATE SENSITIVE PRO-RELIEF (CSPR) 1450 ppm F as MFP and CaCO_3_; (3) COLGATE MAXIMUM CAVITY PROTECTION (CMCP) 1450 ppm F as MFP and CaCO_3_; (4) WHITE GLO SMOKERS FORMULA (WGSF) 1000 ppm F as MFP and CaCO_3_; (5) CEDEL SPEARMINT (CS) 1000 ppm F as MFP and CaCO_3_ and (6) ARM & HAMMER (AH) 1100 ppm F as NaF and calcium sulfate/sodium carbonate (CaSO_4_/Na_2_CO_3_).

**Table 1:** Fluoride type and added amount in calcium-containing dentifrices tested.

Dentifrice	Labelled fluoride compound and concentration	Calcium compound
MI paste one (MPO)^a^	1100 ppm F as NaF	CPP-ACP
Colgate sensitive pro-relief (CSPR)^b^	1450 ppmF as MFP	CaCO_3_
Colgate maximum cavity protection (CMCP)^c^	1450 ppmF as MFP	CaCO_3_
White glo smokers formula (WGSF)^d^	1000 ppmF as MFP	CaCO_3_
Cedel spearmint (CS)^e^	1000 ppmF as MFP	CaCO_3_
Arm and hammer (AH)^f^	1100 ppmF as NaF	CaSO_4_/Na_2_CO_3_

^a^Ingredient list: pure water, glycerol, RECALDENT (CPP-ACP) casein phosphopeptide-amorphous calcium phosphate, sorbitol, CMC-Na, propylene glycol, silicon dioxide, titanium dioxide, xylitol, phosphoric acid, flavoring, methyl salicylate, sodium saccharin, Ethyl p-hydroxybenzoate, propyl p-hydroxybenzoate, butyl p-hydroxybenzoate, sodium-N-lauroyl sarcosinate.

^b^Ingredient list: calcium carbonate, water, sorbitol, arginine, bicarbonate, sodium lauryl sulphate, flavour, sodium monofluorophosphate, sodium silicate, carmellose sodium, sodium bicarbonate, titanium dioxide, acesulfame potassium, xanthan gum, sucralose, limonene.

^c^Ingredient list: calcium carbonate, water, glycerin, arginine, sodium lauryl sulphate, sodium monofluorophosphate, cellulose gum, flavour, sodium bicarbonate, benzyl alcohol, tetrasodium pyrophosphate, sodium saccharin, sodium hydroxide, CI 77891.

^d^Ingredient list: calcium carbonate, aqua (water), glycerin, sorbitol, silica, aroma (flavour), solum diatomeae (diatomaceous earth), sodium lauryl sulphate, carboxymethyl hydroxyethylcellulose, chondrus crispus (carrageenan), CI 77891 (titanium dioxide), sodium saccharin, rosa canina fruit oil, sodium monofluorophosphate.

^e^Ingredient list: water, calcium carbonate, glycerin, aluminium hydroxide, silica, sodium lauryl sulfate, cellulose gum, sodium monofluorophosphate, sodium saccharin, methylparaben, flavours, mineral oil, PEG-60 hydrogenated castor oil, BHA.

^f^Ingredient list: Sodium bicarbonate (baking soda), glycerin, PEG-8, hydrated silica, PEG/PPG-116/66 copolymer, calcium sulphate, sodium lauryl sulfate, aroma, dipotassium phosphate, sodium carbonate, sodium saccharin, cellulose gum, sodium fluoride, limonene, CI 77891.

### Measurement of total and bioavailable F

The methodology for measurement of total and bioavailable fluoride was based on that used by ISO/TC 106 Dentistry SC7 Oral Care Products Working Group 4 Dentifrice. Total F in the dentifrices was measured using a 1:3 water-diluted slurry mixed for 1 min. Bioavailable F was assessed following centrifugation of the slurry and analysis of the supernatant. This laboratory method for the analysis of dentifrice F levels (total and bioavailable) has been widely accepted as a method for simulating normal toothbrushing conditions^22,23,30^. Separation of F followed the Taves microdiffusion method adapted for dentifrices^29^. The dentifrice samples were prepared in triplicate as described below. Four internal standards of 2000, 1500, 1000 and 500 ppm F were formulated with NaF and distilled deionised water (DDW). Dentifrice (10.0 g) was added to 30.0 mL DDW and mixed with a milk frother (Daiso Industries, Hiroshima, Japan) for 1 min. Two 1.0 mL aliquots of the dentifrice slurry were taken, with one immediately centrifuged in a microcentrifuge tube at 12,000 rpm for 2 min. The supernatant of the centrifuged sample was used for bioavailable F analysis, and the uncentrifuged sample used for total F analysis. Samples (0.5 g) of each slurry, supernatant or F standard solution were diluted with 4.50 mL DDW and mixed to homogeneity using a vortex mixer. Diffusion dishes (Bel-Art Conway Diffusion Cell, Sigma Aldrich, MO, USA) for each sample were prepared with petroleum jelly (Vaseline, Unilever Australia, Melbourne Australia) in the outer ring and were placed in an oven at 55 °C for one hour to produce a uniform ring of petroleum jelly. After cooling to room temperature, KOH (0.5 mL of 1 M) was placed in the centre of each diffusion dish, and 3.0 mL of 1 M HClO_4_ saturated with HMDS (2.5 mL HMDS/100 mL HClO_4_) was placed in the middle ring of each diffusion dish. Each dentifrice sample or F standard (0.5 g) was placed in the middle ring of the diffusion dish, but not in contact with the HMDS/HClO_4_ solution also in the middle ring but to one side. The lid was then quickly placed on the diffusion dish to seal with the petroleum jelly outer ring and then the dish was carefully tilted to mix the sample with the HMDS/HClO_4_ solution in the middle ring. A weight was placed on the lid to keep it sealed and the dish was left for 24 h at room temperature.

### Fluoride electrode analyses

A F-ISE (Orion Fluoride Electrode 9609BNWP, Thermo Fisher Scientific) was calibrated using five external F standards of 1, 10, 100, 1000 and 2000 ppm F to confirm correct function of the F-ISE. The standards were prepared by diluting NaF in DDW mixed with equal volume TISAB II solution (Orion ionplus application solution TISAB II, Thermo Fisher Scientific) and were subsequently measured with the F-ISE and an Orion Star A215 PH/ISE Benchtop Meter (Thermos Fisher Scientific). The common logarithm of F concentration (log_10_[F^−^] M) for the external standards was plotted against the EMF (mV) to ensure a linear relationship within the range of − 55 mV to − 60 mV per decade of F concentration in moles per litre. The internal F standards and dentifrice samples from the diffusion dishes were then analyzed using the following procedure. Each diffusion dish lid was gently removed to avoid contamination of the KOH solution in the centre. HCl (0.5 mL of 1 M) was added to the KOH in the centre of each dish and gently mixed followed by the addition of 1.0 mL TISAB II solution. After further gentle mixing, 1.0 mL of the centre solution was removed and placed in a beaker with a small magnetic stirrer and analyzed using the F-ISE. The internal F standards were measured first to plot a calibration curve of the common logarithm of F concentration (log_10_[F^−^] M) against the EMF (mV). The dentifrice samples were then analyzed with the F-ISE and compared against this (internal standard) calibration curve to determine the F concentration.

### Statistical analyses

Means and standard deviations for levels of total F, bioavailable F, percent bioavailable F/total F, and differences between total F and bioavailable F for each dentifrice were calculated. Residuals were assessed for normality using normal Q–Q plots and the Shapiro–Wilk test and homogeneity of variance tested using Levene’s test. The percent total F measured/labelled across the six dentifrices was calculated and differences between dentifrices assessed using the Kruskal–Wallis test with pairwise comparisons using a Bonferroni correction. The measured bioavailable F and the log-transformed percent bioavailable/total F values were compared respectively across the dentifrices using an ANOVA with the Brown–Forsythe statistic and post hoc Dunnett T3 multiple comparison tests for unequal variance. The difference between the total F and bioavailable F for each of the six dentifrices was compared using the paired t-test. All statistical analyses were performed using IBM SPSS Statistics for Windows, version 25 (IBM Corp., Armonk, N.Y., USA) using a significance level of α = 0.05.

## Results

The measured total F and bioavailable F levels of the six calcium-containing, commercial dentifrices are presented in Table 2. All the dentifrices had measured total F concentrations very close to their declared added F concentrations, within a range of approximately 94% to 105% of the total F specified by the manufacturers. The difference between measured total F concentration and respective labelled total F concentrations ranged from 1 to 6%, with no significant differences between dentifrices (p > 0.05). However, the bioavailable F level in each dentifrice ranged from 27 to 97% of the total F added and for all dentifrices, except MPO, the bioavailable F level was significantly lower than the level of F added (Table 2). The MPO dentifrice had the highest bioavailable F (1012 ppm F, representing 97% of the F added), which was significantly higher than the bioavailable F levels of the other five dentifrices (p < 0.05). The CS dentifrice had the lowest bioavailable F level (284 ppm F) and represented only 27% of F added. In addition to having the highest absolute bioavailable F level, MPO had the highest percent bioavailable/total F (97%), which was significantly different to all the other dentifrices tested (p < 0.05).

**Table 2:** Measured total and bioavailable fluoride levels in six calcium-containing dentifrices.

Dentifrice	Total F (ppm) (%total F labelled)	Bioavailable F (ppm)	% Bioavailable/total F	Total F – bioavailable F (ppm)
MPO (1100 ppm F as NaF)	1044.2 ± 12.0 (95%)	1011.8 ± 13.2^abcde^	96.9 ± 0.4^abcde^	32.3 ± 4.4
CSPR (1450 ppm F as MFP)	1486.7 ± 6.7 (102.5%)	903.5 ± 17.7^afghi^	60.8 ± 0.9^afgh^	583.1 ± 11.9^A^
CMCP (1450 ppm F as MFP)	1467.5 ± 6.6 (101.2%)	781.3 ± 12.3^bfjkl^	53.2 ± 0.6^bfi^	686.2 ± 6.6^A^
WGSF (1000 ppm F as MFP)	1040.4 ± 9.9 (104%)	573.7 ± 4.4^cgjmn^	55.1 ± 0.8^cgj^	466.7 ± 12.3^A^
CS (1000 ppm F as MFP)	1054.3 ± 18.5 (105.4%)	283.7 ± 2.9^dhkmo^	26.9 ± 0.3^dhij^	770.6 ± 16.3^A^
AH (1100 ppm F as NaF)	1031.8 ± 69.4 (93.8%)	389.6 ± 14.6^eilno^	37.9 ± 3.5^e^	642.2 ± 79.8^B^
		Overall: p < 0.0001	Overall: p < 0.0001	^A^(p < 0.001)
		^a^p < 0.05	^eg^p < 0.05	^B^(p < 0.01)
		^bcfgjn^p < 0.01	^f^p < 0.01	
		^dhk^p < 0.001	^abc^p < 0.001	
		^eilm^p < 0.0001	^dhij^p < 0.0001	

Values with the same lowercase superscript in columns are significantly different. Values with an uppercase superscript indicate the dentifrice had significantly lower bioavailable F compared with total F.

## Discussion

The microdiffusion method of F analysis was used in the present study to enable accurate measurement of F in a range of dentifrice formulations without interference from formulation ingredients/excipients. The use of internal standards ensured all measurements of samples and standards were from solutions prepared in parallel under the same conditions, maintaining consistency of the analysis. While ionic F can be measured with alternative methods such as ion chromatography, F-ISEs are widely used as they are relatively inexpensive, have low technique sensitivity and high accuracy^31^. However, measurement of soluble F using F-ISEs is known to be difficult in the presence of the dentifrice matrix components^7^. Dentifrice ingredients present in the whole dentifrice slurry or the supernatant, such as detergents and humectants, can poison the electrode. The microdiffusion method involves separation of soluble F through HMDS-mediated HF diffusion and allows analysis of the fluoride without electrode contaminants and regardless of type of F compound^7^. The validity of this technique was evident in the total F measurements reported in the current study, which closely matched the labelled total F for all dentifrices.

All the dentifrices except MPO were measured to have a lower concentration of bioavailable F than their respective total F concentration, hence the null hypothesis was rejected. With the exception of the MPO dentifrice, all dentifrices were observed to have bioavailable F concentrations below 1000 ppm, the concentration deemed significant for caries prevention in permanent teeth^32,33^. In addition to having the highest absolute bioavailable F, the MPO dentifrice had approximately 97% bioavailable/total F, indicating the MPO formulation promoted release of soluble F and the stated F level on the label was an accurate reflection of the dentifrice’s bioavailable F. The relatively low bioavailable F and percent bioavailable/total F observed in the remaining dentifrices may be explained by the type of F and calcium compound in their respective formulations. Of these five dentifrices, four had MFP and CaCO_3_ as ingredients (CSPR, CMCP, WGSF, CS), while the fifth (AH) contained a combination of NaF and CaSO_4_/Na_2_CO_3_, essentially allowing formation of CaCO_3_.

CaCO_3_ is relatively inexpensive compared to other forms of calcium used in dentifrice formulations. It is the most commonly used dentifrice abrasive worldwide, particularly in developing countries where the World Health Organization has encouraged its use for caries prevention in low-cost dentifrice formulations^34,35^. CaCO_3_ and other calcium-based dentifrice additives such as dicalcium phosphate dihydrate (CaH_2_PO_4_·2H_2_O) have been shown to be incompatible with ionic F (e.g. NaF) added to dentifrices^22,36^. In the presence of these calcium-based abrasives, dissociated F ions adsorb onto the poorly soluble calcium carbonate/phosphate phases, and also interact with any calcium ions released from these solid phases to form poorly soluble CaF_2_, thus reducing the bioavailability of F^12,22,37^. In an approach to overcome this loss of bioavailable F, dentifrices containing calcium-based abrasives have been formulated with fluorine covalently bonded to phosphorus in MFP (PFO_3_^2−^). It has been suggested that the MFP molecule does not interact with the poorly soluble calcium solid phases within the dentifrice thus maintaining the intra-oral release of soluble F through saliva/plaque mediated phosphatase hydrolysis of the F-PO_3_ covalent bond^22^. F-ISEs are unable to detect F within MFP, therefore a hydrolysis step (1 M HCl) prior to F-ISE analysis ensures all MFP-bound F is released and identified by the electrode^12^. In the current study, the effectiveness of this hydrolysis step was substantiated by the measured total F of MFP-containing dentifrices closely matching their respective total F levels indicated on the product labels.

In the current study it was evident that following centrifugation of the four MFP-containing dentifrice slurries, a significant proportion of the total F was lost in the precipitate, indicating that F was not bioavailable. Although MFP is suggested to be more compatible with calcium-based abrasives than other F compounds, other investigators have also reported the low bioavailability of F in MFP/CaCO_3_ dentifrice formulations^12,19,38,39^. Over time, spontaneous hydrolysis of MFP within the dentifrice may promote adsorption of soluble F to poorly soluble CaCO_3_ phases prior to product use, particularly with increased temperatures, thus rendering the F inactive^19,22^. Previous studies have indicated MFP-containing dentifrices with calcium-based abrasives can decrease their apparent soluble F to 65% of total F concentration upon aging for 1 year at 22 °C^38,39^. With the very high level of calcium carbonate now added to some dentifrice formulations to help promote remineralization it is also possible that calcium ions (Ca^2+^) are being generated in the dentifrice which can interact with the MFP anion (FPO_3_^2−^) to produce poorly soluble CaFPO_3_. Both these mechanisms would generate poorly soluble F phases and would explain the low bioavailability of F in these dentifrices. The low F bioavailability was found even with the two MFP/CaCO_3_-containing dentifrices with high (1450 ppm F) added F such as CMCP and CSPR where the percent bioavailable/total F concentration was 53% and 61% respectively, and their bioavailable F was considerably less than 1000 ppm F. Similar effects were observed in the other MFP/CaCO_3_-containing dentifrices, with only 55% and 27% of total F in the WGSF and CS dentifrices, respectively, measured as bioavailable. Although the AH dentifrice contained NaF, it also contained CaSO_4_/Na_2_CO_3_ which allowed the formation of poorly soluble CaF_2_ and CaCO_3_. This resulted in poor F solubility and a percent bioavailable/total F of 38%. It was apparent that all dentifrices in the current study containing CaCO_3_ or compounds promoting CaCO_3_ formation had relatively low bioavailable F compared to the total F added, and this was attributed to the adsorption of F onto poorly soluble CaCO_3_ phases or the generation of poorly soluble F phases (CaF_2_ or CaFPO_3_).

Addition of calcium to fluoride containing dentifrices requires a strategy to stabilise the formulation to maintain bioavailability of both ions^25,40^. The MPO dentifrice contained calcium and phosphate ions stabilised in nanocomplexes by casein phosphopeptides (CPP). The CPP are biomimetics of the salivary protein statherin, but they are superior to statherin in their ability stabilise calcium, phosphate and F ions simultaneously, preventing formation of poorly soluble phases and maintaining ion bioavailability through progressive ion release from CPP-ACFP nanocomplexes^41–43^. In a recent study, MPO dentifrice released significantly higher bioavailable calcium and F intraorally compared with other calcium and F-containing dentifrices, and the concentration of bioavailable calcium in tested dentifrices was significantly correlated with enamel remineralization in situ^25^. This demonstrated that the calcium and F ions were stabilised within the MPO dentifrice and were prevented from forming insoluble phases within the dentifrice. This effect was corroborated in the current study, as calcium stabilisation by CPP-ACP in the MPO dentifrice resulted in the highest absolute F bioavailability among dentifrices tested with a percent bioavailable/total F of 97%. Hence, the F level stated on the label of the MPO dentifrice was an accurate representation of its bioavailable F level.

## Conclusions

In summary, a microdiffusion technique successfully measured the total and bioavailable F concentrations of six commercially available dentifrices. Of the six dentifrices, five containing CaCO_3_ were found to have reduced bioavailable F which was attributed to the generation of poorly soluble F phases. The MI PASTE ONE dentifrice contained stabilised calcium as CPP-ACP, promoting F bioavailability which was observed to be 97% of the total F.
